# Investigations on the Effects of Bonding and Forming Conditions on the Deformation Behavior of Copper–Steel Bimetallic Rods during the Cold Drawing Processes

**DOI:** 10.3390/ma17164015

**Published:** 2024-08-12

**Authors:** Yeong-Maw Hwang, Hiu Shan Rachel Tsui, Cheng-Yu Lu

**Affiliations:** 1 Department of Mechanical and Electro-Mechanical Engineering, National Sun Yat-sen University, Kaohsiung 804, Taiwan; ymhwang@mail.nsysu.edu.tw (Y.-M.H.); yukun@nkust.edu.tw (C.-Y.L.); 2 Department of Naval Architecture and Ocean Engineering, National Kaohsiung University of Science and Technology, Kaohsiung 811, Taiwan

**Keywords:** finite element simulation, plastic deformation, residual stress analysis, mechanical properties, drawing process optimization

## Abstract

Metal composite parts are widely used in different industries owing to their significant improvement in material properties, such as mechanical strength, electrical conductivity, and corrosion resistivity, compared to traditional single metals. Such composite parts can be manufactured and processed in different ways to achieve the desired geometry and quality. Among various metal forming techniques, drawing is the most commonly used process to produce long composite wires or rods from raw single materials. During the drawing process of composite wires or rods, not only does the core radius ratio change, but the core or sleeve layer may also undergo necking or fracture due to excessive tensile stresses in the softer layer. In this paper, bimetallic rods with AISI-1006 low-carbon steel cores and C10100 oxygen-free electronic copper sleeves are modeled using the finite element software DEFORM. The simulation models are verified by drawing experiments. The effects of initial bonding conditions, the initial core ratio, reduction ratio, semi-die angle, drawing speed, and friction on the plastic deformation behavior of the bimetallic rods are investigated. The results indicate that the initial bonding conditions have a great impact on the deformation behavior of the billets in terms of strain distribution, material flow, residual stress, and the final core ratio. The permissible forming parameters for obtaining a sound product are investigated as well. With the aid of these analyses, the drawing process and the quality of the products can be controlled steadily.

## 1. Introduction

At present, with the increasing demand for multifunctional materials, traditional single metals can no longer fulfill all the needs of the market. Therefore, various composite parts have been created. One of them is clad rods or bimetallic rods [1]. Bimetallic rods or wires usually have significant improvements in some material properties, such as mechanical strength, electrical conductivity, and corrosion resistivity [2,3,4,5,6], and are required to undergo appropriate forming processes to achieve the required quality and geometry. One of the most commonly used forming techniques is drawing, a stretching process that can produce bars, wires, or tubes with smooth surfaces and precise dimensions. This method is especially suitable for immiscible metals, such as copper and iron, where the solid solution treatment cannot be applied [7].

In past years, some authors have discussed how die geometries and forming conditions would affect the drawn products in a typical drawing process. For example, Haddi et al. [8] stated that the capability of a drawing process depends mainly on the properties of the raw material, the die geometries, and the processing conditions. Tintelecan et al. [9] used 90 sets of steel wire drawing experiments to show that the drawing force was minimized when the semi-die angle was between 6° and 8° and the bearing zone length was around 40% of the product diameter. These findings were confirmed by Martinez et al. [10] in drawing experiments of 99.94% ETP copper wire. Hwang et al. [11] showed that the product diameter of an AISI-316 stainless steel rod increased with the reduction ratio, semi-die angle, drawing speed, and friction and decreased with the bearing zone length of the die.

Recently, several studies have further explored various aspects of the wire drawing process. For instance, Toribio and Lorenzo [12] analyzed the effects of the skin pass technique on residual stress and plastic strain fields in the cold-drawn pearlitic steel wires. Their study revealed that secondary reduction is a key parameter in die design for reducing residual stress and hydrogen accumulation. Hwang [13] investigated the influence of the strain-hardening exponent on temperature and strain distributions during wire drawing, finding that higher strain-hardening rates lead to increased temperature and drawing force, suggesting the need for reduced drawing speed or enhanced cooling. Additionally, Hwang [14] also studied the effects of die angle and strain-hardening rate on microstructure and mechanical properties in wire drawing. The research highlighted that the optimal die angle depends on the strain-hardening rate, with lower angles improving microstructural homogeneity.

This paper is focused on discussing the cold drawing of bimetallic rods with copper sleeves and steel cores. It can be classified into two types: (a) the core and the sleeve are two individual objects with matching dimensions and are in contact with each other, and they are expected to be bonded together as a whole after drawing (hereafter called the unbonded case); (b) the core and sleeve have already been bonded together before entering the die, and the main purpose of drawing is to convert the billet into the required shape and size (hereafter called the bonded case). The unbonded case has been discussed by some researchers. For example, Ragab et al. [15] investigated the deformation of copper–steel and brass–steel bimetal wires during the drawing process and found the relation between the semi-die angle α and core inclination angle β analytically. It was found that when a rod with a hard core and soft sleeve was drawn, the harder constituent suffered nearly no thickness changes, and, hence, β tended to zero. When a rod with a soft core and hard sleeve was drawn, β was slightly smaller than or equal to α. In any case, the value of β should fall between 0 and α, depending on the hardness difference between the two materials. Seet et al. [16] produced micro Ni80Fe20/Cu composite wire with a diameter of 0.096 mm through 66 instances of cold drawing. The drawing experiment began with a fitted Ni80Fe20/Cu billet with a diameter of 3 mm; the reduction ratio of each drawing was 5% and annealing was carried out for every nine draws and after the last drawing. It was found that the sleeve-to-core thickness ratio remained almost unchanged throughout the drawing process and that annealing was essential to release the residual stresses induced by strain-hardening during the process. Malaki et al. [17] showed that removing oxides was an important factor for layer bonding during the cladding process. To achieve a good bonding strength, the surface of the workpieces should first be brushed and cleaned to be free from any undesirable oxide films.

On the other hand, some authors have discussed the bonded case. Szulc et al. [18] studied the drawing process of copper–steel rods and showed that the copper sleeve allowed the rod to obtain a large reduction in cross-sectional area, which cannot be achieved in the conventional drawing of steel; the total reduction after 15 draws without annealing was 93.6%. Ko et al. [19] evaluated the effects of the initial coating (sleeve) ratio, semi-die angle, and reduction ratio on the final coating ratio of copper–steel wire. They assumed the bonding condition to be sticky during the simulations. Both the simulation and experiment results showed that the final coating ratio depended mainly on the initial coating ratio. Chen et al. [20] investigated the plastic deformation behavior of composite aluminum–copper alloy wire as it was drawn through a conical die using finite element simulation. It was found that the composite wire fractured in the neck region under processing conditions of a core ratio of 0.33, reduction ratio of 55%, semi-die angle of 15°, and friction coefficient of 0.3. In all cases, both the maximum damage and maximum effective strain occurred at the interface between the two materials.

Although some work has been carried out to date, few studies have mentioned the influence of initial bonding conditions on the drawing process and drawn products. The present study investigates the drawing of both the bonded and the unbonded cases simultaneously, the deformation behavior of the two cases is compared and discussed, and the effects of various forming conditions on the final core ratio and drawing force are studied, including the initial core ratio, reduction ratio, semi-die angle, drawing speed, and friction between the billet and a die. Through this work, a full picture of the effects of a series of bonding and forming conditions on the cold-drawn copper–steel rods can be established.

## 2. Materials and Methods

DEFORM v11.0.2 is a simulation software that uses finite element analysis to model and analyze metal forming processes. It breaks down the material into small elements, applying equations to predict material response to forces. The software has a vast material behavior library for realistic simulations, considering factors like plasticity and strain rate. DEFORM simulates contact conditions and friction between tools and materials, crucial for accurate material flow and tool wear predictions. With an intuitive interface, it provides detailed data on stress, strain, and potential defects.

**Figure 1:**
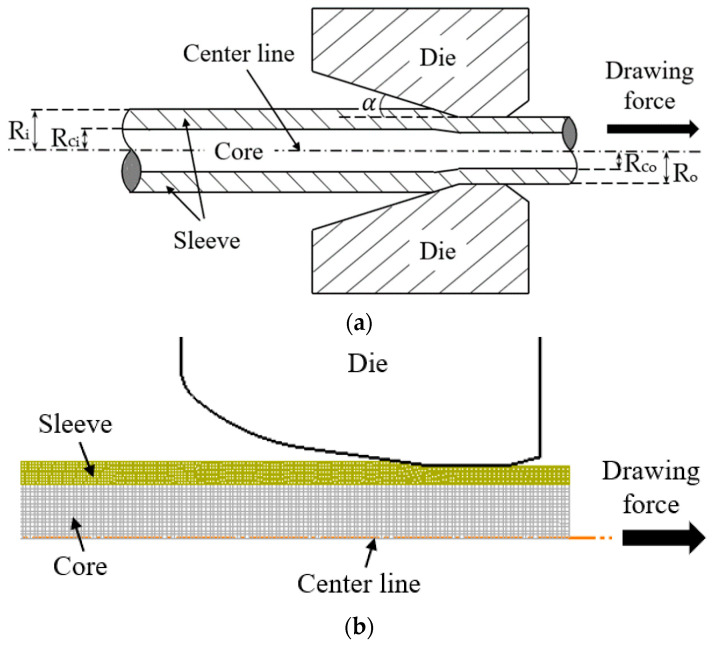
(**a**) Schematic diagram of drawing; (**b**) simulation model of drawing.

The drawing processes were studied through finite element analysis. Bimetallic rods with AISI-1006 carbon steel cores and C10100 oxygen-free electronic copper sleeves were modeled by the finite element software DEFORM using the axial symmetric approach. Figure 1 shows the schematic diagram and the simulation model.

A rigid round die with an exit diameter of 10 mm, a bearing zone length of 5 mm, and a semi-die angle varying from 5° to 9° was modeled. Table 1 and Table 2 show the mechanical properties of the materials and the forming parameters, respectively. The values of Young’s modulus and Poisson’s ratio came from the DEFORM system, and they agree with the typical values of those materials [1,2,3,4,5].

Quadrilateral elements were used and the layer number of elements of the billet in the radial direction was around 20–25 in total, depending on the initial ratio of the sleeve and the core. Since the sleeve deformed more significantly than the core in most cases, denser elements were used in the sleeve, and the element size ratio of the sleeve to the core was 1:10.

## 3. Deformation Behavior of the Rods under Different Initial Bonding Conditions

To investigate the deformation behavior of the rods under different initial bonding conditions, two sets of simulations were carried out. All the forming parameters were the same except for the initial bonding conditions. In the unbonded case, the core and sleeve were assumed to be two individual bodies with matched dimensions and in contact with each other; the friction coefficient between them was 0.3. In the bonded case, the core and sleeve were assumed to be bonded together before entering the die, and a “sticking” boundary condition was set. Another simulation, using a steel rod as the billet, was carried out for comparisons as well. Table 3 shows the forming conditions used in the finite element simulations.

### 3.1. Distribution of Effective Strain during the Drawing Process

**Figure 2:**
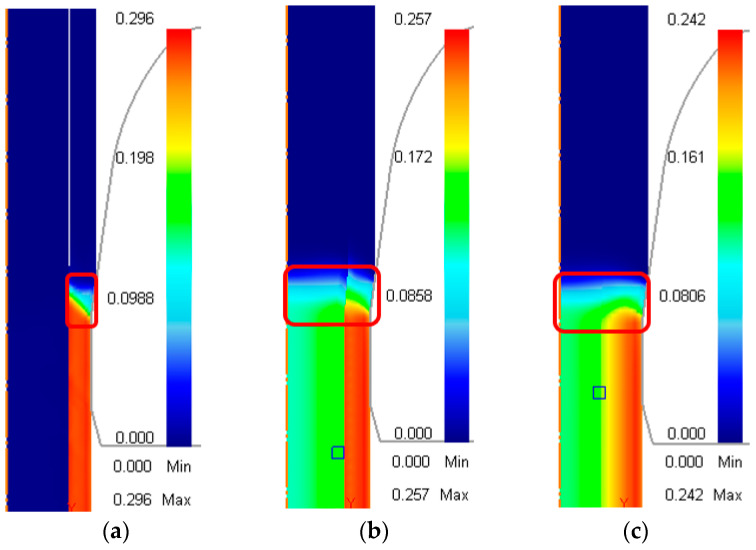
Effective strain distribution of the rods during the steady drawing process. (**a**) Initially unbonded copper–steel rod; (**b**) initially bonded copper–steel rod; (**c**) AISI-1006 steel rod.

Figure 2a–c show the distribution of effective strain during the drawing process in different cases, as listed in Table 3. The rods were drawn downwards by a steady drawing force and the deformation zones are indicated by the red frame. The actual drawing process is conducted on a horizontal surface, as shown in Figure 1. However, for the 2D simulation and result comparison, it is more convenient to present it vertically. 

In the unbonded case (Figure 2a), more than 99% of the deformation occurred in the sleeve, while the dimension of the core was nearly unchanged. For the bonded case (Figure 2b), deformation occurred in both the core and the sleeve; the effective strain in a sleeve was around 2 to 3 times that of the core, and a distinct strain boundary between the core and the sleeve can be observed. The effective strain of the core decreased gradually from the outer part to the middle. These observations agree with the deformation behavior in a typical drawing process, where most deformation occurs near the contact surface with the converging part of the die, as shown in the drawing of steel rods (Figure 2c).

**Figure 3:**
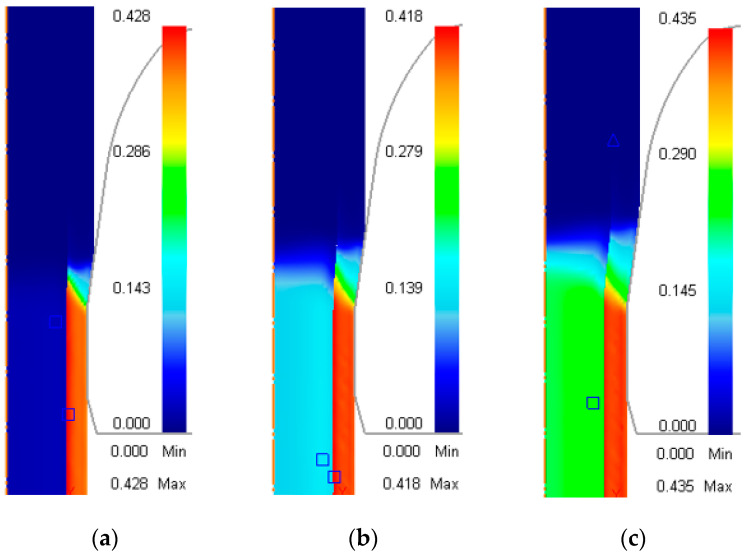
Effective strain distribution of unbonded rods under different reduction ratios: (**a**) 15%; (**b**) 20%; (**c**) 25%.

When the reduction ratio increased to 15% and other forming conditions remained unchanged, as shown in Table 3, the core of the unbonded case started to deform and the extent of deformation increased with the reduction ratio. The reason for this was that the strain-hardening effect of the softer copper sleeve became increasingly significant with an increasing reduction ratio, forcing the steel core to contribute some deformation. Figure 3a–c show the distribution of effective strain at reduction ratios of 15% to 25%.

### 3.2. Material Flow during the Drawing Process

**Figure 4:**
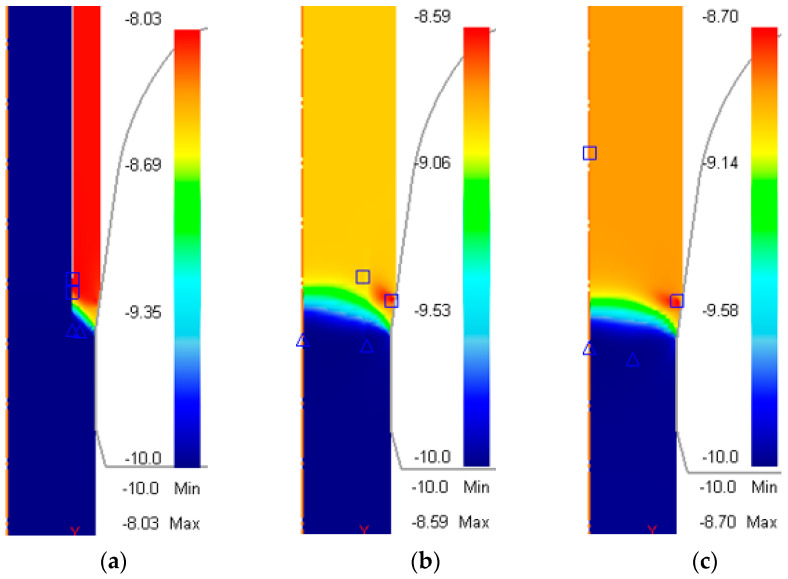
Axial rate distribution (mm/s) of rods during the steady drawing process. (**a**) Initially unbonded copper–steel rod; (**b**) initially bonded copper–steel rod; (**c**) AISI-1006 steel rod.

Figure 4a–c show the axial rate of the materials in different cases as shown in Table 3. In the unbonded case (Figure 4a), the sleeve and the core flowed individually at their own rates before entering the die. The sleeve flowed slower, as it was undergoing elastoplastic deformation near the die entrance, while the core kept the set drawing velocity throughout the drawing process. Both of them had the same axial rate when they entered the bearing zone, as they had already been bonded together. The rate distribution of the bonded case (Figure 4b) was similar to that of the steel rod (Figure 4c); the rod moved forward as a whole and there was no radial rate difference except in the die entrance (the deformation zone).

### 3.3. Distribution of Residual Stress after Drawing

**Figure 5:**
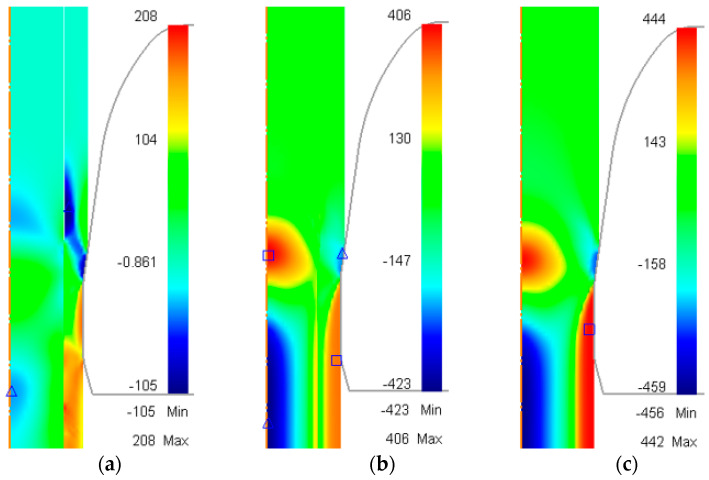
Distribution of axial stress (MPa) during drawing process. (**a**) Initially unbonded copper–steel rod; (**b**) initially bonded copper–steel rod; (**c**) AISI-1006 steel rod.

**Figure 6:**
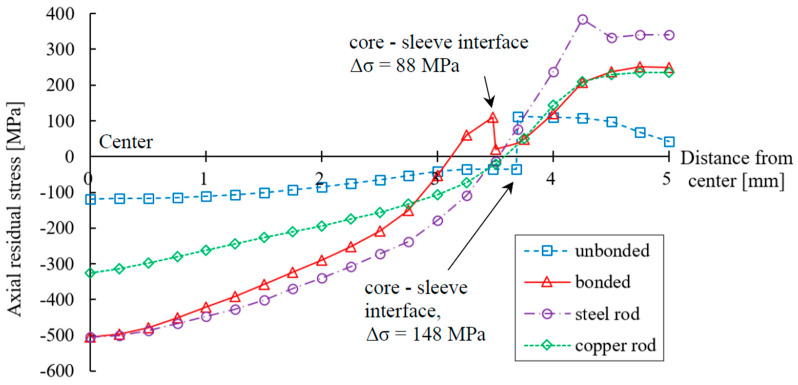
Distribution of axial residual stress after the drawing (reduction ratio of 10%).

Residual stress, accumulated during the drawing process, has a significant impact on the quality of the product, as it is one of the governing factors of crack growth and fatigue [21,22,23]. Figure 5a–c show the distribution of axial stress during the drawing process for the three cases shown in Table 3, while Figure 6 shows the axial residual stress after drawing versus radial distance from the rod center.

An additional simulation, using a copper rod as the billet, was carried out to plot the graph. For the unbonded case, the residual stress in the sleeve was tensile and increased gradually from the rod surface to the core–sleeve boundary; on the other hand, the residual stress in the core was compressive with increasing magnitude from the core–sleeve boundary to the center of the rod; i.e., the largest tensile residual stress was found in the sleeve near the core–sleeve boundary and the largest compressive residual stress was found in the center. The values were 112 MPa and −118 MPa, respectively. 

For the bonded case, the variation of residual stress in the Cu sleeve was similar to that of the Cu rod; the tensile residual stress decreased gradually from 250 MPa at the rod surface to 20 MPa at the core–sleeve boundary. The residual stress in the St core followed the trend in the residual stress curve of the St rod; a tensile stress of 109 MPa was detected near the core–sleeve boundary and decreased gradually until reaching 0 at 3.12 mm from the center. After that, the stress direction changed to compressive and the magnitude kept increasing until reaching the maximum value of −500 MPa in the center. The magnitude of the axial residual stress of the bonded case was always larger than for the unbonded case, except near the core–sleeve boundary.

**Figure 7:**
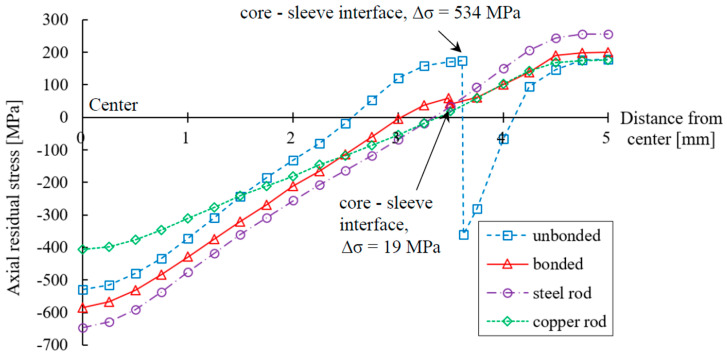
Distribution of axial residual stress after drawing (reduction ratio of 25%).

The simulations were then repeated with the reduction ratio changed to 25%, while other forming conditions remained unchanged as listed in Table 3. The results are shown in Figure 7.

When the reduction ratio increased, the residual stress in the core parts of both the bonded and the unbonded cases became more compressive. For the sleeve, the residual stress for the bonded case became less tensile, whereas in the unbonded case, the residual stress decreased gradually from 178 MPa at the composite rod surface to −360 MPa at the core–sleeve interface. Besides that, as the reduction ratio increased, the change in residual stress at the core–sleeve interface (Δσ) became much larger for the unbonded case and smaller for the bonded case. Fractures may occur when Δσ is too large.

## 4. Effects of the Bonding and Forming Conditions on the Core Ratio of Drawn Products

**Figure 8:**
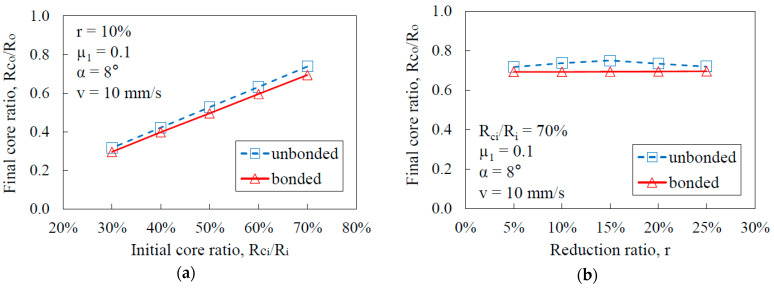

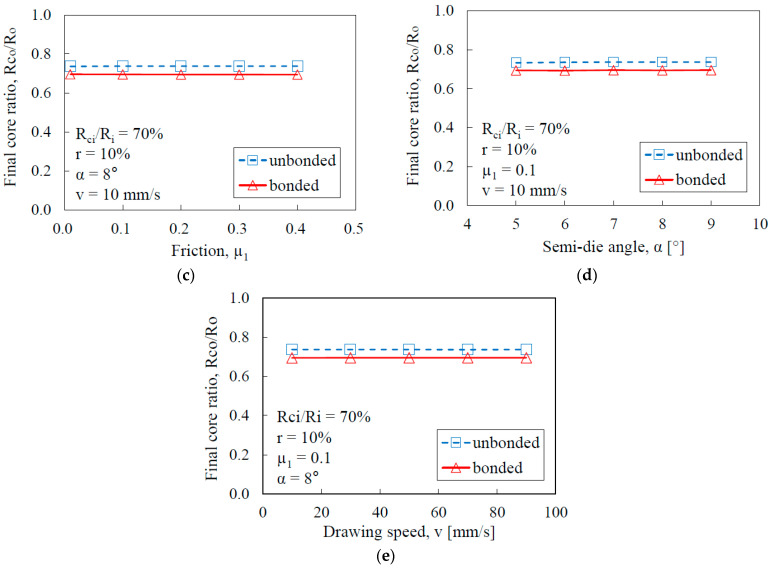
Effects of different forming parameters on the core ratio of drawn products. (**a**) Initial core ratio; (**b**) reduction ratio; (**c**) friction between the die and sleeve; (**d**) semi-die angle; (**e**) drawing speed.

Figure 8a–e show the effects of bonding and forming conditions on the core ratio of drawn products.

From Figure 8a, one can see that the initial core ratio has a significant impact on the final core ratio after drawing. This is because the initial geometry directly determines the distribution of deformation between the core and the sleeve. A higher initial core ratio means that there is more core material relative to the sleeve, which maintains a larger core after drawing due to the concentration of deformation in the sleeve for unbonded cases. Therefore, as the initial core ratio increases, the final core ratio also increases for both bonded and unbonded cases.

In Figure 8b, the reduction ratio does not show a significant effect on the final core ratio for both bonded and unbonded cases. The reduction ratio primarily affects the overall size reduction, rather than the relative proportion of the core to the sleeve. For Figure 8c, the friction between the die and sleeve does not have a significant effect on the final core ratio. Friction influences the force required for drawing, rather than the internal deformation patterns of the materials. In Figure 8d, the semi-die angle also does not significantly affect the final core ratio. The semi-die angle influences the flow of material into the die and the strain distribution. However, the relative deformation between the core and sleeve remains consistent regardless of the semi-die angle, resulting in negligible effects on the final core ratio. In Figure 8e, the drawing speed does not show a significant effect on the final core ratio. Drawing speed affects the rate of deformation and potentially the temperature rise during the process. However, in the range of the speeds tested (10–90 mm/s), these effects are minor and do not substantially alter the relative deformation between the core and sleeve.

Overall, it is clear that the final core ratio depends mainly on the initial core ratio in both the bonded and unbonded cases. For the unbonded cases, the core ratio after the drawing increased by 5–6%, while in the bonded cases, the core ratio after the drawing was nearly the same as the original one. As mentioned in the previous section, the deformation of the unbonded case was more concentrated on the sleeve, compared to the bonded one. Therefore, the final core ratio of the unbonded case was larger than that of the bonded case as a result of less deformation in the core. This phenomenon can be observed under different initial core ratios, reduction ratios, semi-die angles, drawing speeds, and friction levels, as shown in the figures. Hence, one can conclude that the final core ratio of the bonded case is always lower than for the unbonded case under the same conditions.

## 5. Drawing Experiments

Drawing experiments were conducted to verify the simulation results; the experimental conditions are listed in Table 4.

**Figure 9:**
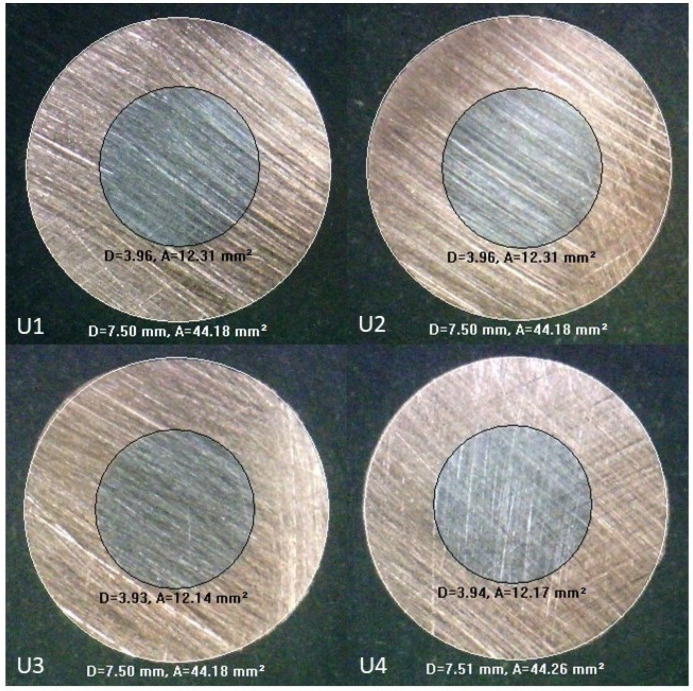
Microscopy images of initially unbonded copper–steel rod after drawing (units: D, diameter [mm]; A, area [mm^2^]).

**Figure 10:**
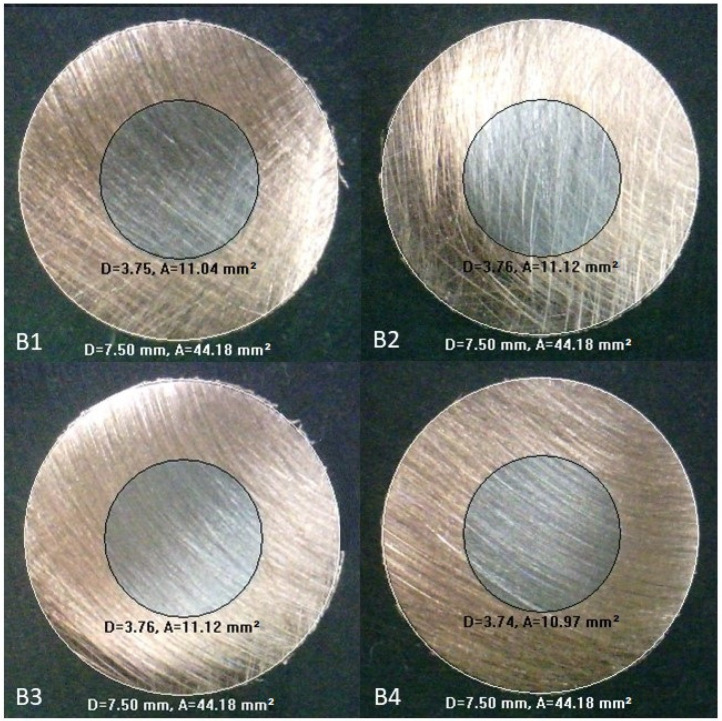
Microscopy images of initially bonded copper–steel rod after drawing (units: D, diameter [mm]; A, area [mm^2^]).

The drawn rods were cut into pieces to obtain the core ratios through the measurement function of the metallographic microscope. Figure 9 and Figure 10 show the microscopy images of the initially unbonded and bonded copper–steel rods, respectively.

The diameters of the steel cores and the whole rods were found using the measurement function of the microscopy system. The core diameters of samples U1 to U4 shown in Figure 9 are 3.96, 3.96, 3.93, and 3.94 mm, respectively, while the rod diameters are 9.50, 9.50, 9.50, and 9.51 mm, respectively. For the initially bonded case shown in Figure 10, the core diameters of samples B1 to B4 are 3.75, 3.76, 3.76, and 3.74 mm, respectively; the rod diameters for the four samples are all 7.50 mm. Table 5 shows the comparisons between the simulation and experimental results; the error in each case is less than 1%, indicating that the simulation models can fit the real situation well.

## 6. Conclusions

The deformation behavior of copper–steel bimetallic rods, for both the bonded and the unbonded cases under different forming conditions, was studied through finite element analysis; the results are summarized below.

1. The deformation of the unbonded case was more concentrated on the sleeve compared to the bonded case under all the tested conditions.
2. The initial bonding condition significantly affected the deformation behavior. In the bonded case, the core and sleeve deformed together, resulting in a more uniform strain distribution.
3. When the reduction ratio increased from 10% to 25%, the axial residual stresses for both the bonded and the unbonded cases became more compressive, and the change in residual stress at the core–sleeve interface became larger for the unbonded case and smaller for the bonded case.
4. The increase in the reduction ratio intensified the strain-hardening effect in the sleeve, particularly in the unbonded case, causing some deformation to be transferred to the core.
5. The core ratio of drawn products depended mainly on the initial core ratio: for the unbonded case, it increased by 5–6% after drawing; for the bonded cases, it was nearly the same as the original one.
6. The simulation and experimental results agreed with each other.
7. Understanding the effects of bonding conditions and forming parameters on deformation behaviors helps in optimizing the cold drawing process for bimetallic rods, guiding industrial applications to achieve better control over product quality and manufacturing efficiency.

## Figures and Tables

**Table 1:** Mechanical properties of materials.

	Die	Sleeve	Core
Material	Tungsten carbide	C-10100 Copper	AISI-1006 Steel
Deformation type	Rigid	Elastoplastic	Elastoplastic
Young’s modulus [GPa]	NA	115	207
Poisson’s ratio	NA	0.33	0.3

**Table 2:** Forming parameters in finite element simulations.

Forming Parameters	Value
Initial outer radius of the composite rod, R_i_ [mm]	5.13, 5.27, 5.42, 5.59, 5.77
Initial core ratio, R_ci_/R_i_, %	30, 40, 50, 60, 70
Reduction ratio, r = (R_i_^2^ − R_o_^2^)/R_i_^2^, %	5, 10, 15, 20, 25
Friction coefficient, µ_1_	0.01, 0.1, 0.2, 0.3, 0.4
Semi-die angle, α [°]	5, 6, 7, 8, 9
Drawing speed, v [mm/s]	10, 30, 50, 70, 90
Initial bonding condition	unbonded, bonded

**Table 3:** Forming conditions of the simulations.

Forming Parameters	Unbonded Case	Bonded Case	Single Steel Rod
Initial outer radius of the composite rod, R_i_ [mm]	5.27	5.27	5.27
Initial core ratio, R_ci_/R_i_, %	70	70	NA
Reduction ratio, r = (R_i_^2^ − R_o_^2^)/R_i_^2^, %	10	10	10
Friction coefficient, µ_1_	0.1	0.1	0.1
Semi-die angle, α [°]	8	8	8
Drawing speed, v [mm/s]	10	10	10
Initial bonding condition	µ_2_ = 0.3	sticking	NA

**Table 4:** Forming conditions of experiments.

Forming Parameters	Value
Initial outer radius of the composite rod, R_i_ [mm]	4.01
Initial core ratio, R_ci_/R_i_	50%
Reduction ratio, r = (R_i_^2^ − R_o_^2^)/R_i_^2^	12.5%
Friction coefficient, µ_1_	0.1
Semi-die angle, α [°]	8
Drawing speed, v [mm/s]	10
Initial bonding condition	unbonded, bonded

**Table 5:** Comparisons between simulation and experimental results.

		Simulations	Experiments	Difference
Initially unbonded case	diameter of the rod	7.50 mm	7.50 mm	0%
	diameter of the core	3.98 mm	3.95 mm	0.76%
	core ratio	53.07%	52.67%	0.76%
Initially bonded case	diameter of the rod	7.50 mm	7.50 mm	0%
	diameter of the core	3.76 mm	3.75 mm	0.27%
	core ratio	50.13%	50.00%	0.27%

## Data Availability

The original contributions presented in the study are included in the article, further inquiries can be directed to the corresponding author.
